# Measuring athletic performance in post-metamorphic fire salamanders

**DOI:** 10.1186/s13104-021-05808-0

**Published:** 2021-10-26

**Authors:** Erica de Rysky, Bisconti Roberta, Chiocchio Andrea, Canestrelli Daniele

**Affiliations:** Dipartimento Di Scienze Ecologiche E Biologiche, Università Della Tuscia. Viale Dell’Università S.N.C, 01100 Viterbo, Italy

**Keywords:** Athletic performance, Fire salamander, *Salamandra salamandra*, Swimming

## Abstract

**Objective:**

Athletic performances are dynamic movements that are physically challenging and often predict individual success in ecological contexts. They stem from a complex integration of multiple phenotypic traits—*e.g*., morphological, physiological and behavioural—that dictate animal survival and individual fitness. However, directly quantifying athletic performances can be particularly challenging in cryptic, slow-moving species or not very reactive in attitude. Here we present and describe a rapid, simple, and low-cost method to measure athletic performance in post-metamorphic individuals of the fire salamander *Salamandra salamandra*. While extremely reactive during the larval stage, adult salamanders are, in fact, cryptic and relatively slow-moving.

**Results:**

Forcing terrestrial juveniles to swim under standard, albeit ecologically plausible, laboratory conditions, and using an automatic point-mass tracking tool, we were able to measure maximal and average performance indicators of post-metamorphic individuals. This method avoids inter-individual variation in motivation, as it forces individuals to perform at their best. Moreover, with this method, measures of athletic performance will be directly comparable between larval and terrestrial stages, allowing to study the contribution of carryover effects to the wide range of processes implicated in the eco-evo-devo of athletic performance in salamanders.

**Supplementary Information:**

The online version contains supplementary material available at 10.1186/s13104-021-05808-0.

## Introduction

Athletic performances are key abilities in animals’ life since they predict individual success in ecological contexts [1, 2]. They consist of dynamic movements that are physically challenging, allowing animals to interact with other conspecific and heterospecific organisms, and with the physical environment [1–3]. These abilities stem from a complex integration of multiple phenotypic traits – *e.g.*, morphological, physiological and behavioural –, that dictate animal survival and fitness in a wide range of contexts [2–4]. Consequently, the comparative study of maximal individual performances in athletic tasks can offer an excellent window on the diversity and evolution of functional phenotypic traits within and among species, and on the role of athletic abilities in moulding the evolutionary pathways of intra- and interspecific lineages in response to significant changes of their physical and biotic environment [5–8].

Among athletic abilities, locomotor performance has been extensively studied, due to its direct implication in a wide range of ecological tasks, such as predator escape, prey capture, territory defence, reproductive success, dispersal, and so forth [1, 3, 6, 9, 10]. Traditionally, the bulk of studies investigating the evolution of locomotor traits have been focused on analysing the origin (*e.g.*, convergent vs independent) and the evolutionary trends in animal locomotion along the tree-of-life [1 and reference therein]. However, more recently, increasing attention is being focused on studying inter-individual differences in locomotor performances within and among populations within species [11–13]. In fact, there is an increasing awareness that athletic abilities can contribute to shaping the eco-evolutionary processes implicated in the genesis of biogeographic patterns both above and below the species level [14–16]. Among vertebrates*,* much research effort has been devoted to the study of athletic abilities in the lizards of the genus *Anolis* [17–19] and cane toad *Rhinella marina* [15, 20–23]. The integrative analysis of jumping performances in these study systems (based on biomechanical, physiological, and morphometric traits; [22, 24]) highlighted an unexpected amount of inter-individual diversity in performance abilities and suggested a major role of these abilities in shaping the evolution both of dispersal capacities and the species range, over multiple spatial and temporal scales [19, 22, 23, 25]. As a result, increasing insights on the eco-evolutionary role of inter-individual differences in animal performances traits are coming from this kind of studies. However, performing experimental studies can be in many cases hampered by species-specific lifestyles, such as in species that are cryptic and tend not to react impulsively to external *stimuli*. In these species, identifying and directly quantifying genuinely informative athletic performance traits could be challenging.

**Fig. 1:**
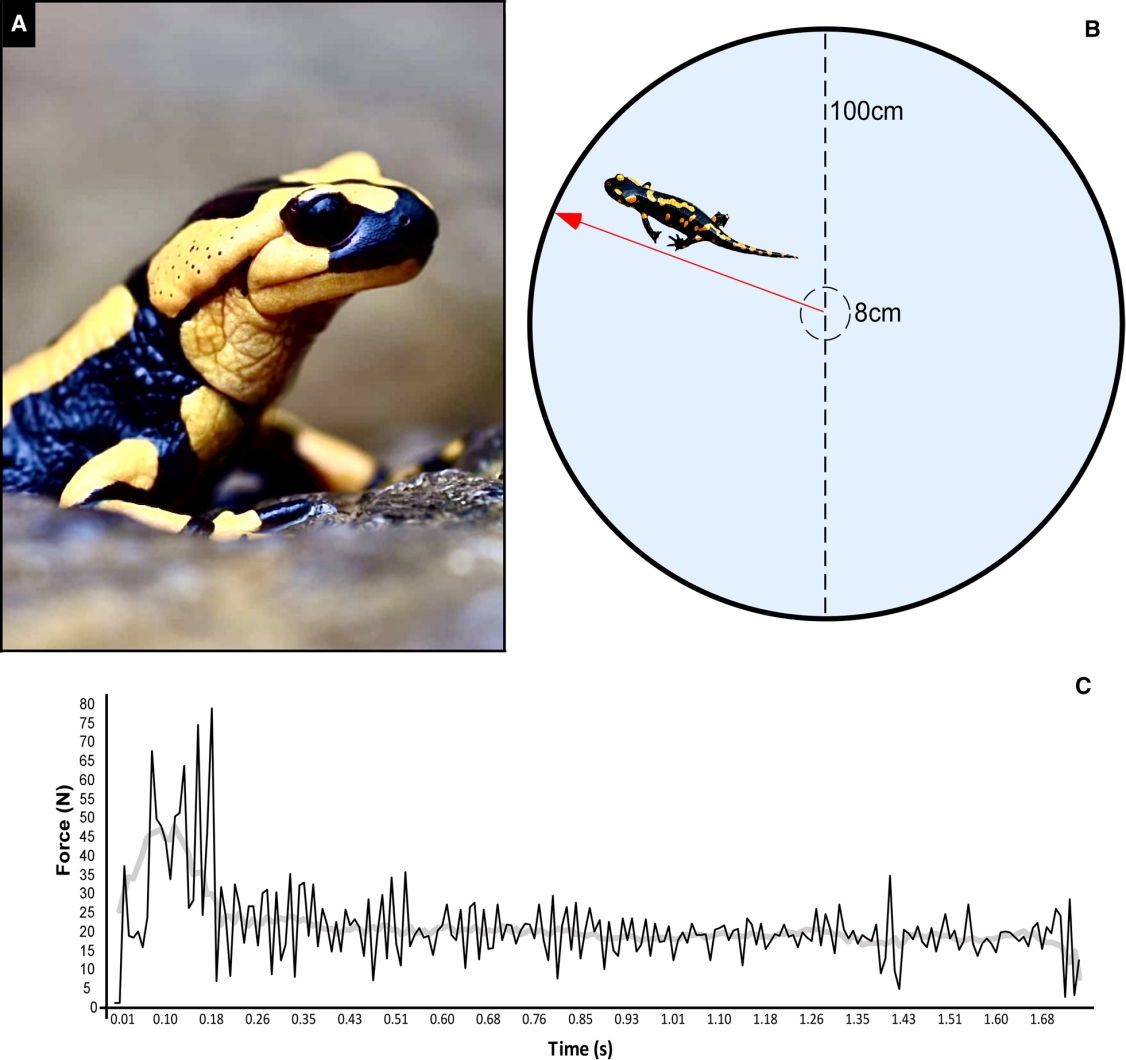
**A** The fire salamander *Salamandra salamandra* (Photo: Grignani G.). **B** The arena used for the swimming test (salamander is not in scale). **C** Swimming force profile of an individual as a representative example (black line: raw data; grey line: moving average)

The European fire salamander *Salamandra salamandra* (Fig. 1A) is a urodele amphibian widely distributed across much of the Western Palaearctic, where it inhabits mixed, moist deciduous forests from the sea level up to 2500 m [26, 27]. It is a nocturnal and crepuscular dweller, mostly active on rainy nights, spending daytime under moisty woods, stones or the leaflitter [26]. After the metamorphosis, the fire salamander becomes strictly terrestrial, and only females approach water to lay larvae. In the adult phase, this species shows a distinctively cryptic lifestyle, making the study of maximal locomotor performance quite difficult. Therefore, when found above ground its movements are slow and, even when stimulated by a (natural or mimicked) predator, its defensive behaviour is rather static, characterized by remaining motionless, usually without attempting to escape predator attacks (it possesses antipredator toxin secretions) [28–31]. Nevertheless, measuring maximal locomotor performances is of crucial importance because they are directly implicated in the survival of individuals for prey capture, reproductive success, and dispersal ability.

Here we present a simple, rapid and low-cost method to accurately quantify athletic performance abilities in post-metamorphic fire salamanders. We aim to provide a test that could force this cryptic, and apparently unreactive species, to perform at its extreme limit, allowing the analysis of athletic performance to become part of the extensive body of literature concerning patterns of phenotypic diversity, distribution, and evolution in this species [32, 33].

## Main text

### Methods

#### Sampling and housing

We collected 78 fire salamander individuals at larval stage immediately after deposition to standardise the entire experimental procedure. With this aim, we monitored breeding sites in the Picentini Mts. (Campania Region, Italy) between March and June in 2019, when the larvae laying usually occurs [27]. Larvae were collected from distinct breeding sites placed at least 20–50 m from each other, in order to avoid kinship relationships among individuals. All the breeding sites consisted in small ponds along small streams characterized by low-slope and low water velocity/turbulence located in beech forests at around 1000 m of altitude. Collected larvae were individually housed under controlled and standard conditions [34] in a humid chamber (room temperature 18–20° C, humidity 65–70%, natural photoperiod: 12–15 h daylight/dark following natural seasonality) at the facilities of the Department of Ecological and Biological Sciences of the University of Tuscia (Viterbo, Lazio, Italy). Salamander larvae were individually housed within pierced plastic baskets (10 × 10 × 10 cm), each with a terracotta saucer as shelter. All plastic baskets were placed collectively within a PVC tank filled with aerated soft water (dechlorinated and demineralized water 1:1) and water temperature (10–12 °C) monitored using a temperature data logger (Hobo Pendant MX2201). Larvae were fed ad libitum three days a week with live tubifex and chironomid larvae. Once metamorphosed, each salamander was housed individually into terrariums made with transparent and micro-perforated plastic boxes (11.5 × 11.5 × 6 cm), with coconut litter and gravel as substrates, a plastic cap containing dechlorinated water, and a piece of cork bark as shelter. Metamorphosed juveniles were fed ad libitum with crickets (*Acheta domestica*) and mealworms (*Tenebrio molitor*).

#### Athletic performance test

A convenient approach to analyse athletic performance in animals consists of measuring maximal movement performance in response to a *stimulus* that is perceived as threatening, usually cues of a predator attack [*e.g.*, 35–37]. We performed a series of pilot tests on a group of non-experimental animals (10 individuals) in an attempt to elicit propelling movements. However, even the more invasive version of these tests (i.e., tail pinching) did not elicit consistent and repeatable responses. Most individuals did not react at all, while a few others jumped, u-turned, or sprinted, and the responses were not repeatable over multiple trials. Based on that result, we moved to test individual response to another common threat in fire salamander environments: falling into the water. As mentioned above, adult fire salamanders are fully terrestrial. Accidental falls into the water may be fatal, unless the shore is reached promptly. Preliminary trials revealed a consistent response of salamanders to swimming challenges, which is necessary to reflect individual differences.

Swimming performance was measured once for each individual at three months after the metamorphosis to ensure the performance being representative of a fully terrestrial life stage. Swimming tests were carried out under strictly controlled environmental conditions (room temperature 20 °C, humidity 70%; water temperature 15 °C) since variability in these conditions might impact individual performance during the test.

The experimental arena consisted of a white circular plastic tank (diameter: 100 cm; water volume: 50L; water depth: 6.5 cm; see Fig. 1B) to avoid possible “blind spots”, where individuals may get stuck or have a foothold to climb out of the water. To standardise the starting phase of the test, when the individuals were released in the tank, the centre of the arena was marked with a small plastic circle placed 1 cm above the water surface. Thus, each individual was gently dropped into the water from the centre of this “launchpad”, using a humid spoon. Test duration was set to 2 min. At the end of the test, individuals were weighted with a precision analytical scale (± 0.001 g) to calculate the acceleration force. All individuals were fasted 48 h before the tests.

Swimming performance tests were video recorded at high frame rates (120 fps), using a GoPro Hero5 camera (at 1080p) placed above the centre of the experimental arena. Videos were then analysed with the software Tracker 5.1.3 (https://physlets.org/tracker). This software allows characterising physical properties of animal movements using automatic point-mass tracking, if high-quality videos are used as input (i.e., enough contrast between tracked objects and the background), together with a measure of the body mass and a known reference scale. We used the tip of the salamander snout as a point mass for tracking and manually set the body mass for each individual and calibrate the scale by setting the diameter of the tank as reference scale. Tracker allows extracting a wide range of performance variables. For illustrative purposes, here, we provide maximum and average values of velocity and acceleration. Potential errors in automatic tracking were checked by eye for  each individual by the same operator.

### Results and discussion

**Fig. 2:**
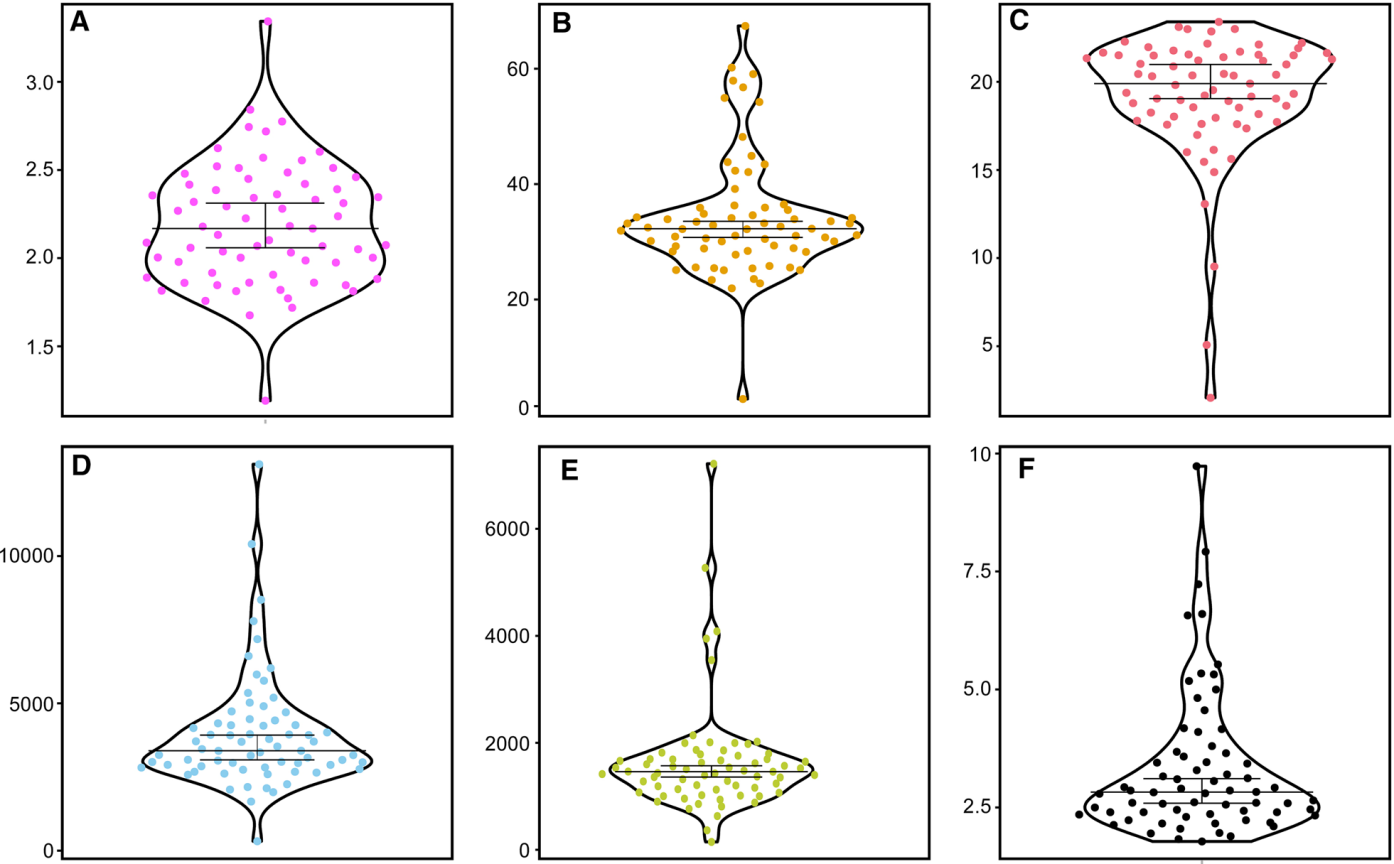
Violin plots of the measured variables. Horizontal lines display the mean value, and vertical bars show the 95% confidence interval. **A** Weight (g); **B** Max speed (cm/s); **C** Mean speed (cm/s); **D** Max swimming force (N); **E** Mean swimming force (N); **F** Time to reach the edge (s). The graphs were drawn using the web app PlotsOfData [44] and the software Canvas 11 (ACD Systems of America, Inc.)

All the analysed individuals started swimming immediately after the dive, and continued swimming on the water surface until they reached the edge of the tank (Fig. 1C; Table 1; Fig. 2; an additional movie file shows this in more detail [see Additional file 1]). This behavioural pattern has at least two highly desirable features when testing athletic performances: (1) complete inter-individual consistency; (2) a bi-axial configuration of the entire athletic movement. Consistency of the behavioural pattern is essential, as it implies that individual personality does not modulate an individual’s tendency to perform the athletic task at its best, and so it does not act as a confounding experimental factor [2, 38–40]. This argument receives further support by observing that maximum acceleration values are usually reached at the beginning of the test, which is not expected in case personality (*e.g.*, shy vs bold response to a stressful situation) play a significant role affecting motivation. Furthermore, the bi-axial configuration of the movement over the water surface allows ignoring the third (i.e., vertical) axis when deriving acceleration and other physical descriptors of the athletic performance, simplifying the experimental setup substantially.

**Table 1:** Maximum (Max) and mean values of speed and acceleration, and mean time to reach the edge of the experimental arena, measured for the post-metamorphic individuals of *Salamandra salamandra* analysed

Variable	Mean ± S.E
Max speed (cm/s)	34.21 ± 1.63
Mean speed (cm/s)	19.19 ± 0.45
Relative max speed (g/s)	0.07 ± 0.01
Relative mean speed (g/s)	0.13 ± 0.01
Max swimming force (N)	3949.81 ± 237.23
Mean swimming force (N)	1628.21 ± 125.63
Mean time to reach the edge (s)	3.68 ± 0.39

Once individuals reached the edge of the tank, the behavioural pattern changed significantly, towards a heterogeneous alternation of resting at the edge, swimming and floating phases. Moreover, over multiple testing, consistency of the pattern was observed neither among nor within individuals, which qualifies this second segment of the test as useless for our purposes. It is worth noting that this second segment implies that endurance measures cannot reliably be derived from this test, and the test duration could be reduced from 2 to 1 min for future use. At the same time, however, this diverse mix of multiple phases might reveal distinct behavioural types along the second segment of the test. If and to what extent behavioural patterns along this segment (*e.g.*, frequency and/or time spent in each phase) might associate with individual personality traits could be an intriguing subject for future experimental scrutiny, given the inherent difficulties in studying personality traits variation in highly cryptic and rather static species.

Measuring animals’ athletic performances in cryptic, slow-moving, or difficult-to-track animals could be very challenging [1]. Here, we have described a rapid, simple, and low-cost method to measure athletic performance in fire salamanders under standardized, albeit environmentally realistic, laboratory conditions. By forcing individuals to perform in the aquatic environment, this method successfully avoids the lack of motivation, in line with results obtained with similar approaches in other organisms (*e.g.,* in rodents, [41, 42]). Finally, a point of significant value of the approach presented here stems from the possibility of gaining comparable athletic performance measures between larval and terrestrial stages. Indeed, when in the aquatic medium, post-metamorphic salamanders show a swimming pattern matching the one used by larvae. In light of this, our test will allow us to investigate the contribution of carryover effects across life stages to the wide range of processes implicated in the eco-evo-devo of athletic performance in salamanders [43].

## Limitations

- The method does not allow to measure the endurance of locomotor effort.
- The automatic tracking of the focal subject is only applicable when high-quality videos are used as input (i.e., enough contrast between tracked objects and the background).

## Supplementary Information

**Additional file1:** Video-tracking of the swimming performance test of a post-metamorphic fire salamander *Salamandra salamandra*, obtained by using Tracker 5.1.3.

## Data Availability

The datasets used during the current study available from the corresponding author on reasonable request.
